# Residential inequality and spatial patterns of infant mortality in Ethiopia: evidence from Ethiopian Demographic and Health Surveys

**DOI:** 10.1186/s41182-021-00299-y

**Published:** 2021-01-27

**Authors:** Getayeneh Antehunegn Tesema, Achamyeleh Birhanu Teshale

**Affiliations:** Department of Epidemiology and Biostatistics, Institute of Public Health, College of Medicine and Health Sciences, University of Gondar, Gondar, Ethiopia

**Keywords:** Infant mortality, Spatial patterns, Ethiopia

## Abstract

**Background:**

Despite the remarkable decrease in infant mortality rate in most countries, the rate of decline is slow and it remains unacceptably high in Sub-Saharan Africa. The progress in infant mortality in Ethiopia is far below the rate needed to achieve the Sustainable Development Goal. Understanding the residential inequality and spatiotemporal clusters of infant mortality is essential to prioritize areas and guide public health interventions. Therefore, this study aimed to investigate the residential inequality and spatial patterns of infant mortality in Ethiopia.

**Methods:**

A secondary data analysis was done based on the Ethiopian demographic and health surveys conducted in 2000, 2005, 2011, and 2016. A total weighted sample of 46,317 live births was included for the final analysis. The residential inequality was assessed by calculating the risk difference in infant mortality rates between urban and rural live births and presented using a forest plot. For the spatial patterns of infant mortality, the SaTScan version 9.6 and ArcGIS version 10.6 statistical software were used to identify the spatial patterns of infant mortality.

**Results:**

The study revealed that the infant mortality rate significantly declined from 96.9 per 1000 live births [95% CI 93.6, 104.2] in 2000 to 48.0 per 1000 live births [95% CI 44.2, 52.2] in 2016 with an annual rate of reduction of 3.2%. The infant mortality rate has substantial residential inequality over time, which is concentrated in the rural area. The spatial distribution of infant mortality was significantly clustered at the national level in survey periods (global Moran’s I, 0.04–0.081, *p* value < 0.05). In 2000, the most likely clusters were found in east Afar and at the border areas of south Amhara and north Oromia regions (LLR = 7.61, *p* value < 0.05); in 2005, at the border areas of Southern Nations Nationalities and People and in the entire Amhara region (LLR = 10.78, *p* value< 0.05); in 2011, at Southern Nations Nationalities and People and Gambella regions (LLR = 6.63, *p* value< 0.05); and in 2016, at east Oromia and northeast Somali regions (LLR = 8.38, *p* value < 0.05).

**Conclusion:**

In this study, though infant mortality has shown remarkable reduction, infant mortality remains a major health care concern and had significant spatial variation across regions. Besides, the study found that infant mortality was highly concentrated in rural areas. Identifying the hotspot areas of infant mortality would help in designing effective interventions to reduce the incidence of infant mortality in these areas. Therefore, the findings highlighted that public health interventions should target rural areas and identified hotspot areas to reduce the incidence of infant mortality.

## Background

Globally, 7.2 million children died during the first year of birth, accounting for 73% under-five mortality [1]. Nearly 80% of infant mortality is caused by preventable causes [2, 3]. Since 1990, the global child mortality rate has decreased from 65 per 1000 live births to 29 per 1000 live births in 2017 [4]. Despite the dramatic decrease in the worldwide infant mortality rate, low-income countries continue to share the largest burden of infant mortality [4, 5].

Infant mortality is a major global public health concern especially in Sub-Saharan Africa (SSA) [6–8]. It is considered an important national indicator of health because it is particularly sensitive to the socio-economic development and basic living conditions of the country [9]. However, remarkable progress has been made by many countries to achieve the Millennium Development Goal 4 (MDG 4) to reduce child mortality by two thirds between the years 1990 and 2015 [10]; half of the world’s nations are still behind their targets [11]. In Ethiopia, the infant mortality rate has declined from 123 in 1990 to 48 in 2015 with a huge residential and regional disparity [12]. The rate of reduction was below the MDG-4 target of 67% reduction [13, 14]. It is highly concentrated in rural residents and emerging regions of Ethiopia (Beneshangul Gumuz, Afar, Gambela, and Somali regions) [12, 15].

Previous literatures documented that advanced maternal age, maternal education, wealth status, antenatal care (ANC) utilization, parity, birth order, place of delivery, vaccination, and residence were significant predictors of infant mortality [16–20]. Besides, a spatial study done in South Africa showed that infant mortality had a spatial variation across the country and the hotspot areas of infant mortality were detected in socially and economically marginalized populations [17].

Though there are studies reported on the prevalence and associated factors of infant mortality in Ethiopia, as to our search of the literature, there is limited evidence on the residential inequality and spatial patterns of infant mortality in Ethiopia. Investigating the residential inequality and spatial patterns of infant mortality is crucial in evaluating the effectiveness and impact of implemented public health programs. Moreover, identifying the hotspot areas of infant mortality would help public health planners, policymakers, programmers, and partners to design effective strategies and interventions to reduce infant mortality. Therefore, this study aimed to investigate the residential inequality and spatial patterns of infant mortality in Ethiopia.

## Methods

### Data source and sampling procedure

A secondary data analysis was done based on the four consecutive Ethiopian Demographic and Health Surveys (EDHSs) conducted in 2000, 2005, 2011, and 2016. These EDHSs are nationally representative surveys conducted in Ethiopia every 5-year interval to generate updated health and health-related indicators. The EDHSs were done on the nationally representative sample at the regional level as well by rural and urban areas. The enumeration areas (EAs) were selected with probability proportional to the population size of the strata, and standard questionnaires were used to collect the data. Ethiopia is located in the Horn of Africa, and it has nine regions and two city administrations. More than 80% of the country’s total population lives in the regional states of Amhara, Oromia, and Southern Nations Nationalities and Peoples Region (SNNPR) [21]. An EA is a geographic area that covers an average of 181 (104–324) households. The sampling frame contains information about the EA location, type of residence (urban or rural), and the estimated number of residential households. In all EDHSs, a two-stage stratified cluster sampling technique was employed to select the study participants using the 1994 Population and Housing Census (PHC) for EDHS 2000 and 2005, and the 2007 PHC for EDHS 2011 and 2016 as a sampling frame. In the first stage, 539 EAs for EDHS 2000, 540 EAs for EDHS 2005, 624 EAs for EDHS 2011, and 645 EAs for EDHS 2016 were selected. At the second stage, on average, 28 to 32 households per cluster were systematically selected. A total weighted sample of 46,317 (12,260 in EDHS 2000, 11,163 in EDHS 2005, 11,872 in EDHS 2011, and 11,022 in EDHS 2016) infants were included for analysis. The detailed sampling procedure was presented in the full EDHS report [14, 22–24].

### Study variables

#### Outcome variables

The outcome was infant death, which is the death of a live-born infant in the first year of life. In this analysis, it was recorded as a binary variable. Death of an infant within the first year of age was coded as 1, and 0 if the child was alive at the time of the survey.

### Data collection procedure

The EDHS data was accessed from the DHS program’s official database (www.measuredhs.com) through an online request. The raw data was collected in all parts of the country using a structured and pre-tested questionnaire [14, 22–24]. We used the Kids Record (KR) dataset. The geographic coordinate data (longitude and latitude) were taken at the cluster level/enumeration area level.

### Data management and analysis

The data were weighted using sampling weight, primary sampling unit, and strata before any statistical analysis to restore the representativeness of the survey and to get reliable statistical estimates. Descriptive and summary statistics were done using the STATA version 14 software.

### Residential inequality

The urban-rural difference in infant mortality across regions over time was assessed using a forest plot. In the forest plot, a risk difference (RD) with a 95% confidence interval (CI) was reported to declare the presence of significant residential inequality in infant mortality rate across regions. An RD greater than 0 showed that the infant mortality rate is highest in rural areas whereas the risk difference less than 0 indicates the infant mortality rate is higher in urban areas. A risk difference of 0 indicates there is no difference in infant mortality across the residence.

### Spatial analysis

For the spatial analysis, the ArcGIS version 10.6 and Kuldorff’s Spatial Scan Statistical analysis (SaTScan) version 9.6 statistical software were used to identify the hotspot areas of infant mortality.

The spatial autocorrelation (Global Moran’s I) statistic was done to assess whether the spatial distribution of infant mortality was dispersed, clustered, or randomly distributed in the study area [25]. Moran’s I is a spatial statistic used to measure spatial autocorrelation by taking the entire dataset and produce a single output value that ranges from − 1 to + 1. Moran’s I values close to − 1 indicate infant mortality dispersed, whereas Moran’s I values close to + 1 indicate infant mortality clustered, and disease distributed randomly if the I value is zero.

In SaTScan analysis, the Bernoulli-based model was employed to identify statistically significant spatial clusters of infant mortality using Kuldorff’s SaTScan version 9.6 software. The SaTScan uses a circular scanning window that moves across the study area. A child who died during the first year of life was taken as a case, and children who were alive were taken as controls to fit the Bernoulli model. The numbers of cases in each location had Bernoulli distribution, and the model required the cases, controls, and geographic coordinates data. The default maximum spatial cluster size of < 50% of the population was used, as an upper limit, which allowed both small and large clusters to be detected and ignored clusters that contained more than the maximum limit.

For each potential cluster, a likelihood ratio test statistic and the *p* value were used to determine if the number of observed infant mortality cases within the potential cluster was significantly higher than expected or not. The scanning window with maximum likelihood was the most likely performing cluster, and the *p* value was assigned to each cluster using Monte Carlo hypothesis testing by comparing the rank of the maximum likelihood from the real data with the maximum likelihood from the random datasets. Based on the log-likelihood ratio (LLR) values, the spatial window that has the largest LLR value is considered as primary clusters (more likely clusters), and the remaining significant clusters are considered as secondary clusters. The primary and secondary clusters were identified and assigned *p* values and ranked based on their likelihood ratio test, based on 999 Monte Carlo replications [26].

The spatial interpolation was employed to predict infant mortality on the unsampled areas in the country based on the sampled EA values. There are various deterministic and geostatistical interpolation methods. Among all of the methods, ordinary Kriging and empirical Bayesian Kriging are considered the best methods since it incorporates the spatial autocorrelation and it statistically optimizes the weight [27]. The ordinary Kriging spatial interpolation method was used for predictions of infant mortality in unobserved areas of Ethiopia since it had the smallest root mean square error (RMSE) and residual.

### Ethical approval and consent to participate

This study was a secondary data analysis of existing publicly available EDHS data. Permission for data access was obtained from measure demographic and health survey through an online request from http://www.measuredhsprogram.com. The geographic coordinate data were obtained by explaining the purpose of using GPS data, and we receive approval from the Measure DHS program.

## Results

### Descriptive results

A total of 46,317 infants were included in this study. From 2000 to 2011, the percentage of urban residents rose from 10.4 to 12.9%, and the percentage of women who did not obtain formal education declined from 82.1 to 66.1% from 2000 to 2016. Besides, the proportion of media exposure showed a small rise in the four EDHS surveys, from 27.1% in 2000 to 33.1% in 2016. The proportion of children born to Orthodox Christian followers declined from 49.3% in 2000 to 34.2% in 2016, while the number of children born to Muslim religious followers rose from 30.3% in 2000 to 41.4% in 2016. Regarding maternal age, there is a slight decrement in the percentage of infants born to mothers aged 15–19 years in the last 16 years (from 4.6 to 3.4%). According to child nutritional status, the percentage of severely stunted and underweight infants increased from 19 to 23.6% and 12.2 to 17% in the last 16 years, respectively. Whereas, the percentage of severely wasted infants decreased from 8.3 to 6.2% from 2000 to 2016. Over the last 16 years, the percentage of the optimal birth interval (≥ 24 months) decreased from 84.1 to 82.4% (Table 1). The overall infant mortality rate significantly decreased from 96.9 per 1000 live births [95% CI 93.6, 104.2] in 2000 to 48 per 1000 live births [95% CI 44.2, 52.2] in 2016, with an annual reduction rate (ARR) of 3.2%.

**Table 1:** Percentage distribution of characteristics of respondents in 2000, 2005, 2011, and 2016 Ethiopian Demographic and Health Surveys

Variables	EDHS 2000 (%) (*N* = 12,260)	EDHS 2005 (%) (*N* = 11,163)	EDHS 2011 (%) (*N* = 11,872)	EDHS 2016 (%) (*N* = 11,022)
**Region**				
Tigray	788 (6.4)	698 (6.3)	753 (6.3)	716 (6.5)
Afar	126 (1.0)	107 (1.0)	121 (1.0)	114 (1.0)
Amhara	3202 (26.1)	2621 (23.5)	2656 (22.4)	2072 (18.8)
Oromia	4999 (40.8)	4411 (39.5)	5014 (42.2)	4851 (44.0)
Somali	142 (1.2)	477 (4.3)	364 (3.1)	507 (4.6)
Benishangul	124 (1.0)	105 (0.9)	140 (1.2)	122 (1.1)
SNNPRs	2602 (21.2)	2500 (22.4)	2494 (21.0)	2296 (20.8)
Gambella	29 (0.2)	31 (0.3)	40 (0.3)	27 (0.2)
Harari	25 (0.2)	22 (0.2)	29 (0.2)	26 (0.2)
Addis Ababa	182 (1.5)	153 (1.4)	221 (1.9)	244 (2.2)
Dire Dawa	40 (0.3)	37 (0.3)	39 (0.3)	47 (0.4)
**Place of residence**				
Urban	1276 (10.4)	815 (7.3)	1528 (12.9)	1215 (11.0)
Rural	10,984 (89.6)	10,348 (92.7)	10,344 (87.1)	9807 (89.0)
**Religion**				
Orthodox	6042 (49.3)	4674 (41.9)	4519 (38.1)	3772 (34.2)
Catholic	81 (0.7)	121 (1.1)	108 (0.9)	103 (0.9)
Protestant	1959 (16.0)	2217 (19.9)	2758 (23.2)	2329 (21.1)
Muslim	3713 (30.3)	3875 (34.7)	4214 (35.5)	4561 (41.4)
Traditional	465 (3.8)	275 (2.5)	271 (2.3)	257 (2.3)
**Age of women (in years)**				
15–19	557 (4.6)	575 (5.2)	492 (4.1)	378 (3.4)
20–24	2750 (22.4)	2243 (20.1)	2360 (19.9)	2068 (18.8)
25–29	3297 (26.9)	3171 (28.4)	3798 (32.0)	3353 (30.4)
30–34	2433 (19.8)	2301 (20.6)	2344 (19.7)	2489 (22.6)
35–39	1839 (15.0)	1718 (15.4)	1822 (15.4)	1772 (16.1)
40–44	991 (8.1)	787 (7.1)	785 (6.6)	723 (6.6)
45–49	393 (3.2)	367 (3.3)	272 (2.3)	239 (2.2)
**Maternal education**				
No education	10,062 (82.1)	8838 (79.2)	8227 (69.3)	7284 (66.1)
Primary	1597 (13.0)	1855 (16.6)	3211 (27.1)	2950 (26.8)
Secondary	573 (4.7)	427 (3.8)	266 (2.2)	514 (4.7)
Higher	28 (0.2)	43 (0.4)	168 (1.4)	274 (2.5)
**Height/age**				
Normal	7644 (62.4)	9149 (82.0)	6893 (58.1)	6624 (60.1)
Moderately stunted	2292 (18.7)	980 (8.8)	2258 (19.0)	1802 (16.4)
Severely stunted	2324 (19.0)	1034 (9.2)	2721 (22.9)	2596 (23.6)
**Weight/age**				
Normal	7937 (64.7)	9487 (85.0)	7288 (61.4)	6998 (63.5)
Moderately underweight	2837 (23.1)	1192 (10.7)	2615 (22.0)	2146 (19.5)
Severely underweight	1486 (12.2)	484 (4.3)	1969 (16.6)	1879 (17.0)
**Weight/height**				
Normal	11,091 (90.5)	10,701 (95.7)	9896 (83.4)	8931 (81.0)
Moderately wasted	1025 (8.3)	368 (3.3)	758 (6.4)	686 (6.2)
Severely wasted	144 (1.2)	94 (0.8)	1218 (10.3)	1406 (12.8)
**Media exposure**				
No	8932 (72.9)	7017 (62.9)	6988 (58.9)	7375 (66.9)
Yes	3328 (27.1)	4146 (37.1)	4884 (41.1)	3647 (33.1)
**Preceding birth interval**				
< 24 months	1950 (15.9)	2246 (20.1)	1963 (16.5)	1942 (17.6)
≥ 24 months	10,310 (84.1)	8917 (79.9)	9909 (83.5)	9080 (82.4)
**Parity**				
Less than 4	6821 (55.6)	6139 (55.0)	6893 (58.1)	6270 (56.9)
5 up to 8 births	4291 (35.0)	4027 (36.1)	4039 (34.0)	3999 (36.3)
Greater than 8 births	1148 (9.4)	997 (8.9)	940 (7.9)	753 (6.8)

### Residential inequality in infant mortality rate

**Fig. 1:**
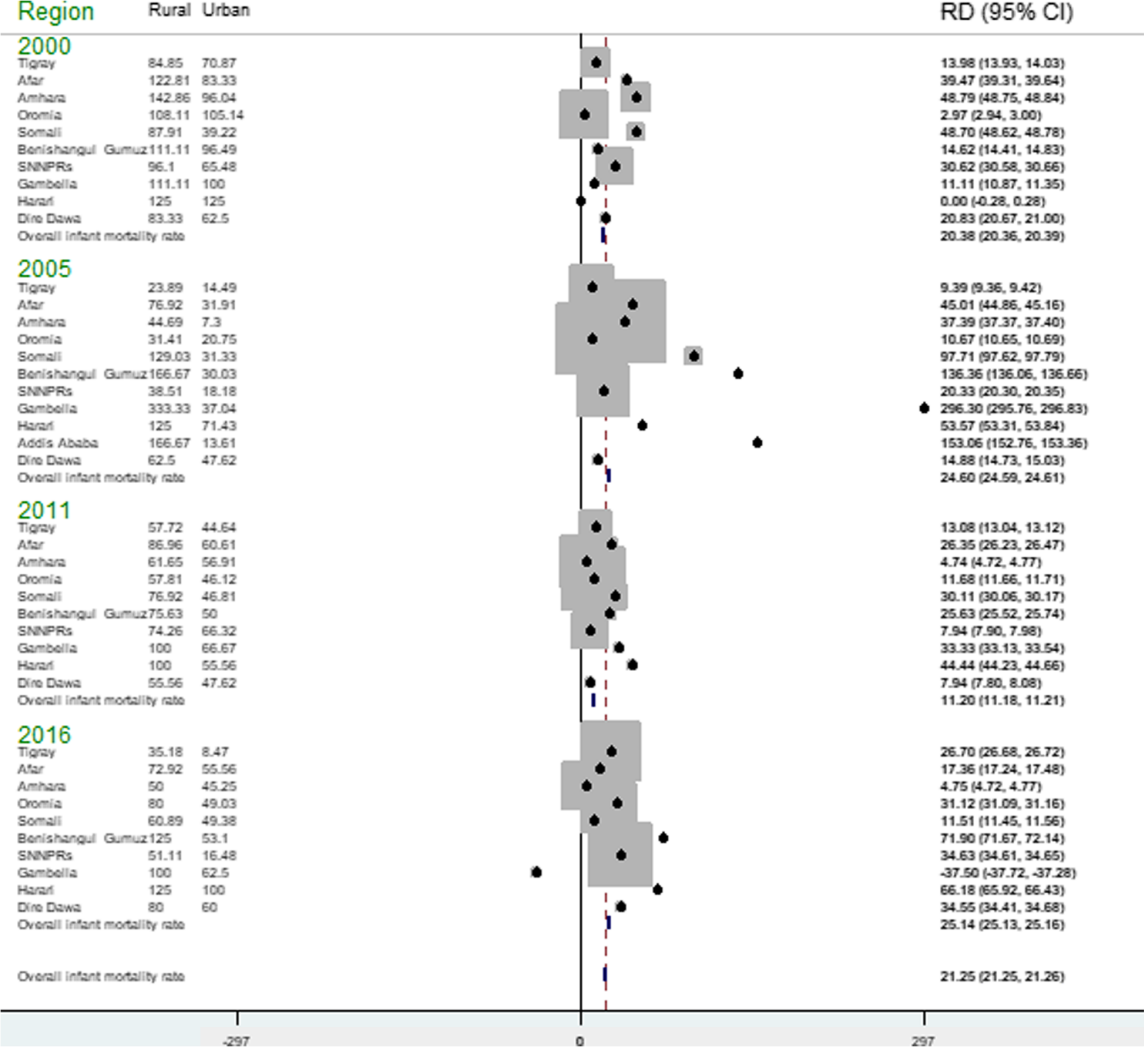
Forest plot of urban-rural risk difference of infant mortality across regions in Ethiopia over time

The infant mortality rate has shown significant residential inequality. The residential inequality in infant mortality rate slightly increased from 20.38 per 1000 live births in 2000 to 21.25 per 1000 live births in 2016. In EDHS 2000, overall, there was a statistically significant residential difference in IMR (RD = 20.10, 95% CI 20.08, 20.11) with the highest residential inequality observed in the Somali region which was (RD = 48.69, 95% CI 48.61, 48.77) followed by the Amhara region (RD = 46.82, 95% CI 46.77, 46.87). In EDHS 2005, overall, there was a statistically significant residential inequality in IMR (RD = 24.60, 95% CI 24.59, 24.61) with the highest urban-rural inequality observed in the Gambella region (RD = 296.29, 95% CI 295.75, 296.83) while the lowest was observed in the Oromia region (RD = 10.66, 95% CI 10.64, 10.68). In EDHS 2011, there was a statistically significant residential difference in infant mortality rate (RD = 11.20, 95% CI 11.18, 11.21) with the highest residential inequality in infant mortality observed in Harari regions (RD = 44.44, 95% CI 44.23, 44.65) while the lowest risk difference in the Amhara region (RD = 4.72, 95% CI 4.71, 4.74). In EDHS 2016, there was a substantial residential inequality in the infant mortality rate in Ethiopia (RD = 25.15, 95% CI 25.14, 25.16) with the highest residential inequality observed in the Benishangul Gumuz region (Fig. 1).

### Spatial patterns of infant mortality

The spatial patterns of infant mortality were found non-random at all the study periods (global Moran’s I value of 0.061 (*p* < 0.05) in EDHS 2000, 0.062 (*p* < 0.0001) in EDHS 2005, 0.081 (*p* < 0.01) in EDHS 2011, and global Moran’s 0.08 (*p* < 0.05) in EDHS 2016), indicating that there was a significant clustering of infant mortality in Ethiopia.

**Fig. 2:**
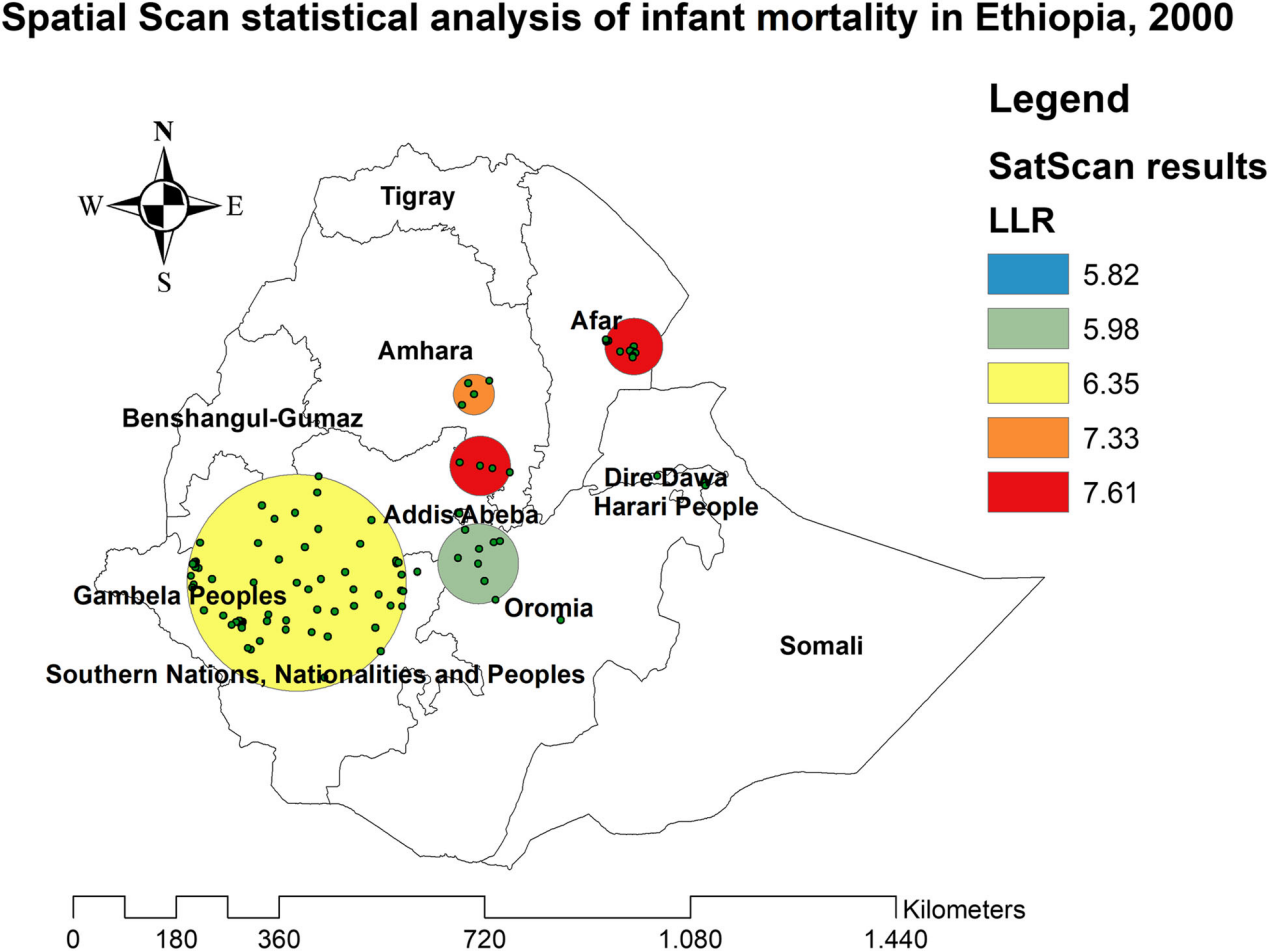
The SaTScan analysis of infant mortality in Ethiopia in 2000

**Fig. 3:**
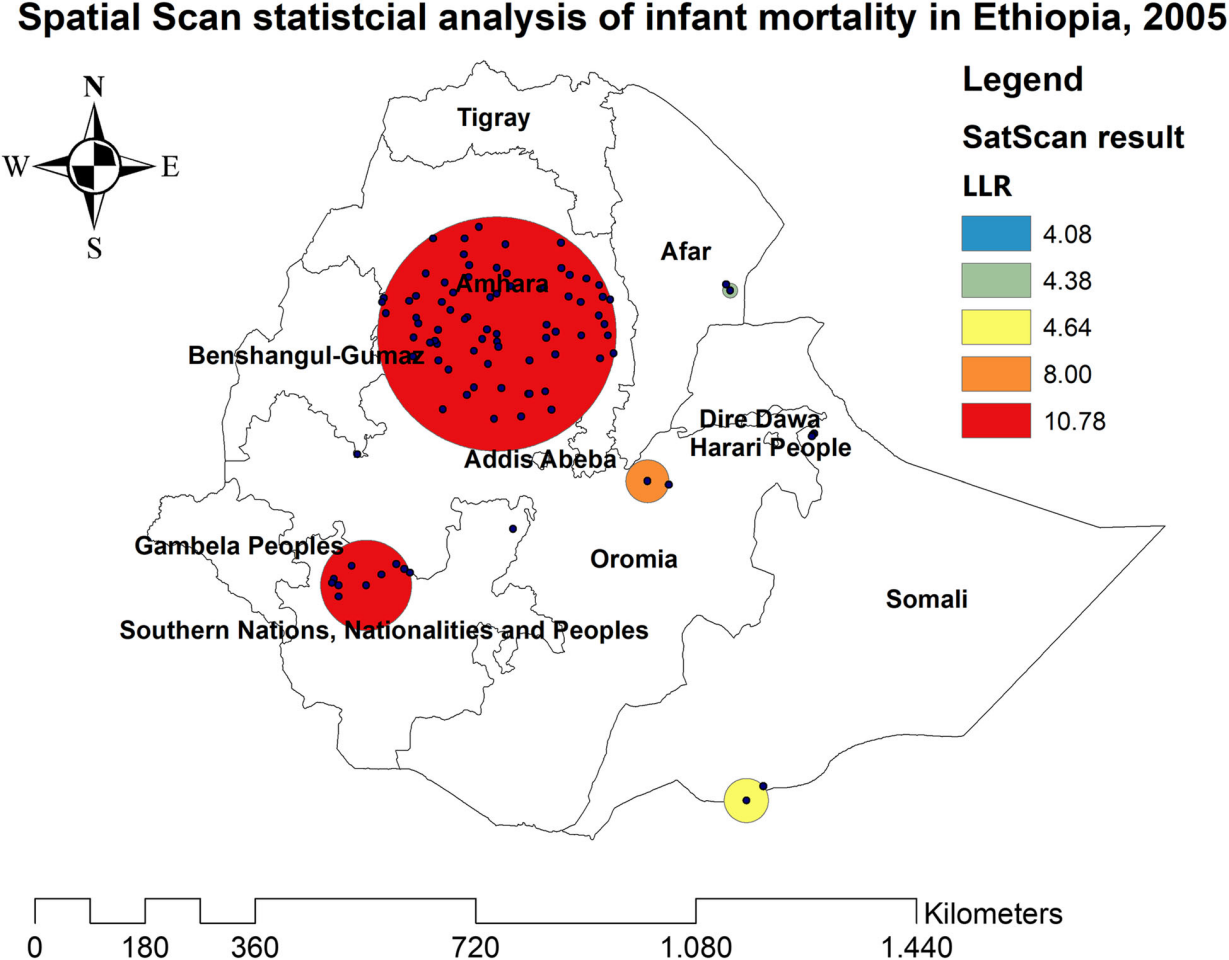
The SaTScan analysis of infant mortality in Ethiopia in 2005

**Fig. 4:**
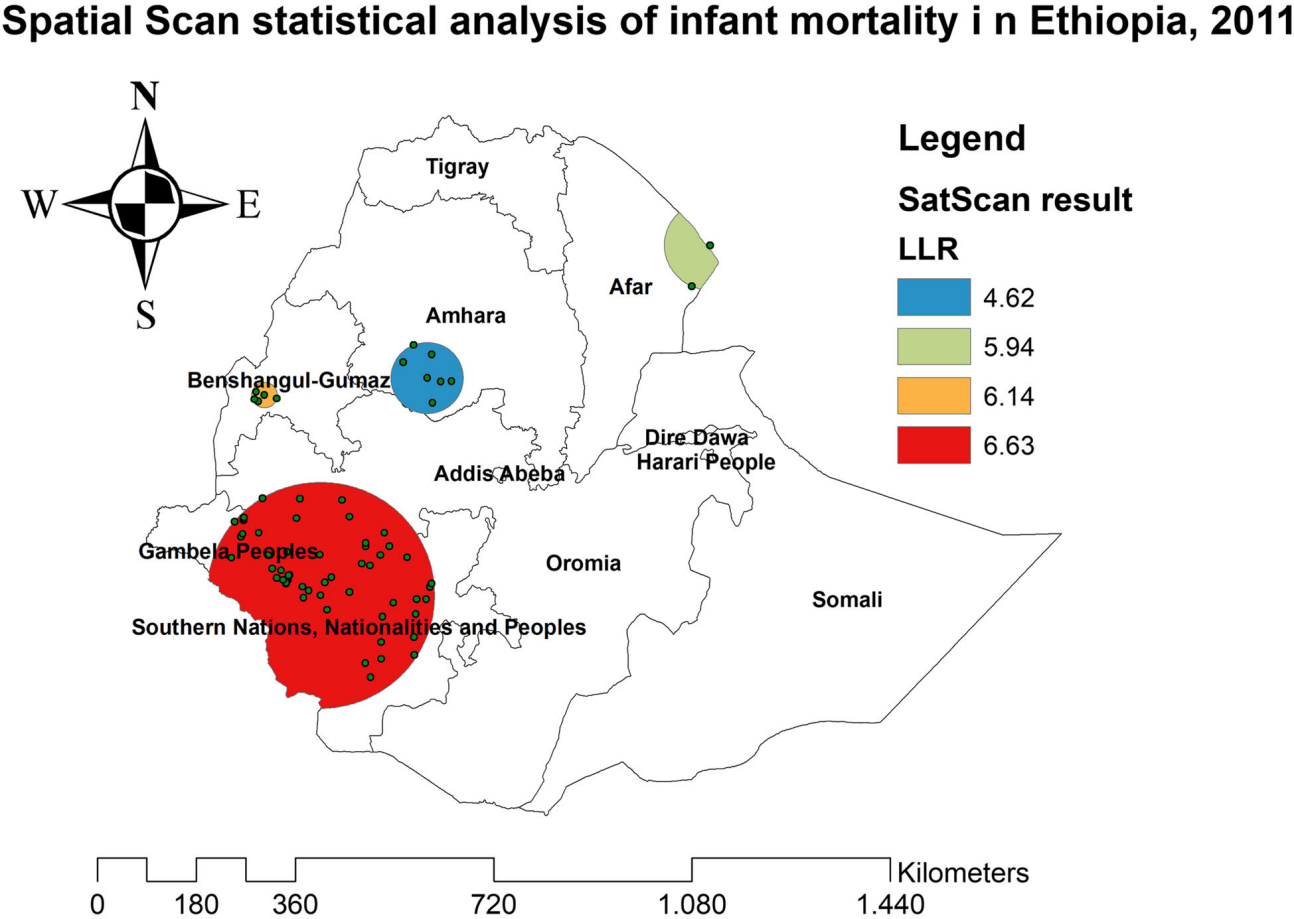
The SaTScan analysis of infant mortality in Ethiopia in 2011

**Fig. 5:**
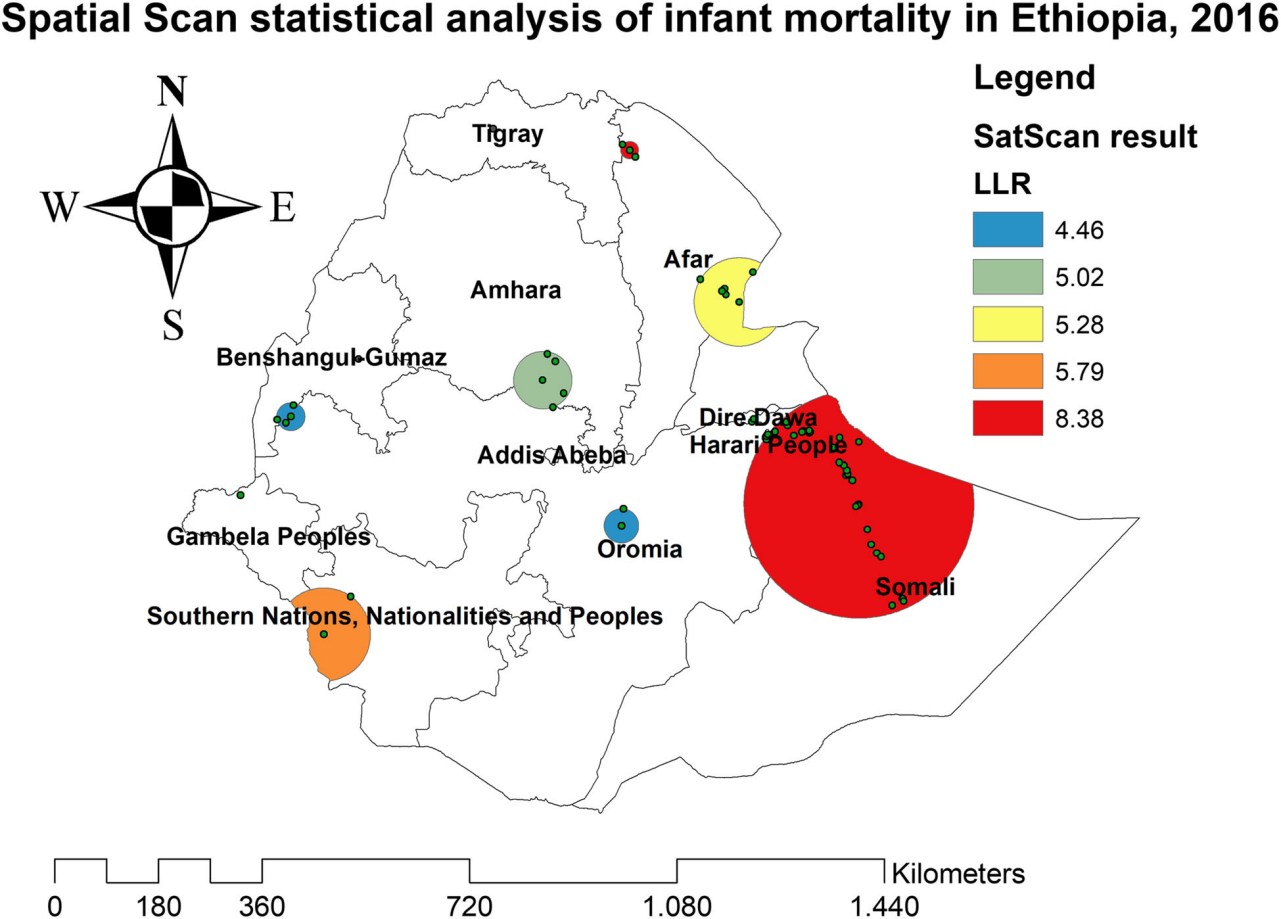
The SaTScan analysis of infant mortality in Ethiopia in 2016

In the spatial scan statistical analysis, a total of 162 significant clusters (12 clusters in 2000, 82 clusters in 2005, 60 clusters in 2011, and 38 clusters in 2016) were identified in all four surveys. Of the total significant clusters, 143 clusters were primary clusters and 19 clusters were secondary clusters. In 2000, the most likely clusters of infant mortality were found at the border areas of north Oromia and Amhara, and east Afar regions of Ethiopia. The cluster’ spatial window was centered at 13.228089 N, 39.395499 E with a 45.01-km radius, and LLR of 7.61, at *p* < 0.05. Infants within the spatial window were 2.24 times (RR = 2.24) more likely to die than infants outside the spatial window (Fig. 2 and Table 2). In 2005, the most likely clusters of infant mortality were located in the entire Amhara and north Oromia regions of the country. The cluster’s spatial window was centered at 10.829534 N, 38.136563 E with a 191.38-km radius, with a RR of 1.55 and LLR of 10.78, at *p* < 0.01. Infants within the spatial window were 1.55 times (RR = 1.55) more likely to die than infants outside the spatial window (Fig. 3 and Table 3). In 2011, the most likely clusters of infant mortality were identified in the entire Gambella and Southern Nations Nationalities and Peoples regions of Ethiopia. The cluster’s spatial window was centered at 7.001534 N, 35.851801 E with a 205.31-km radius and LLR of 6.63, at *p* < 0.01. Infants within the spatial window were 1.49 times (RR = 1.49) more likely to die than infants outside the spatial window (Fig. 4 and Table 4). In 2016, the most likely clusters were detected in the east Oromia and southeast Somali regions of Ethiopia. The cluster’s spatial window was centered at 8.222771 N, 43.571949 E with a 197.67-km radius and LLR of 8.38, at *p* < 0.01. Infants within the spatial window were 5.21 times (RR = 5.21) more likely to die than infants outside the spatial widow (Fig. 5 and Table 5).

**Table 2:** Spatial scan statistical analysis of hotspot areas of infant mortality in Ethiopia, 2000

Cluster	Enumeration area (cluster) identified	Coordinate/radius	Population	Case	RR	LLR	*p* value
1 (8)	37, 50, 49, 35, 34, 33, 36, 38	(13.228089 N, 39.395499 E)/45.01 km	174	4	2.24	7.61	< 0.01
2 (4)	121, 196, 195, 157	(9.764568 N, 39.076488 E)/52.22 km	103	23	2.35	7.33	< 0.05
3 (12)	51, 54, 52, 55, 53, 56, 57, 81, 61, 60, 59, 58	(11.644629 N, 41.494678 E)/49.58 km	204	36	1.86	6.36	0.627
4 (25)	427, 431, 435, 436, 438, 437, 439, 443, 440, 423, 447, 441, 450, 452,442, 434, 422, 448, 449, 453, 444, 451, 445, 432, 426	(9.294072 N, 42.169897 E)/7.91 km	342	16	0.48	5.98	0.757
5 (22)	270, 271, 234, 233, 266, 243, 231, 232, 230, 350, 268, 378, 276, 349,347, 379, 382, 267, 348, 346, 228, 345	(3.781487 N, 39.231315 E)/310.10 km	531	30	0.57	5.82	0.81

**Table 3:** Spatial scan statistical analysis of hotspot areas of infant mortality in Ethiopia, 2005

Cluster	Enumeration area (cluster) identified	Coordinate/radius	Population	Case	RR	LLR	*p* value
1 (72)	338, 99, 461, 307, 182, 415, 396, 304, 354, 351, 479, 264, 17, 27, 149, 231, 211, 74, 511, 1, 244, 364, 115, 181, 350, 322, 97, 463, 418, 152, 326, 447, 214, 212, 98, 24, 192, 458, 125, 288, 156, 15, 187, 215, 270, 296, 110, 225, 427, 483, 262, 239, 75, 102, 218, 349, 22, 402, 96, 250, 29, 159, 101, 278, 145, 11, 128, 190, 56, 516, 289, 157	(10.829534 N, 38.136563 E)/191.38 km	1348	142	1.55	10.78	0.007
2 (10)	184, 266, 474, 355, 507, 299, 421, 533, 336, 387	(7.138868 N, 36.215549 E)/73.74 km	245	36	2.06	8.00	0.04
3 (2)	290, 33	(8.668135 N, 40.349713 E)/34.77 km	72	13	2.53	4.64	0.846
4 (1)	394	(9.062726 N, 36.087008 E)/0 km	27	7	3.57	4.38	0.908
5 (2)	416,449	(3.976226 N, 41.801891 E)/36.02 km	43	9	2.88	4.07	0.95

**Table 4:** Spatial scan statistical analysis of hotspot areas of infant mortality in Ethiopia, 2011

Cluster	Enumeration area (cluster) identified	Coordinate/radius	Population	Case	RR	LLR	*p* value
1 (55)	409, 457, 207, 243, 315, 389, 364, 477, 559, 327, 639, 631, 223, 368, 511, 404, 160, 107, 545, 520, 125, 30, 598, 135, 354, 259, 277, 390,283, 105, 586, 610, 642, 580, 239, 267, 405, 301, 427, 552, 530, 342,475, 396, 375, 395, 252, 273, 339, 206, 242, 116, 357, 130, 383, 434	(7.001534 N, 35.851801 E)/205.31 km	1160	101	1.49	6.63	< 0.01
2 (5)	6, 625, 276, 100, 157	(10.274364 N, 34.932796 E)/23.02 km	129	19	2.43	6.14	< 0.05
3 (2)	604,512	(12.721978 N, 42.213619 E)/80.78 km	47	10	3.50	5.95	0.463
4 (7)	90, 18, 484, 43, 398, 439, 635	(10.553829 N, 37.591523 E)/64.57 km	125	17	2.24	4.63	0.878

**Table 5:** Spatial scan statistical analysis of hotspot areas of infant mortality in Ethiopia, 2016

Cluster	Enumeration area (cluster) identified	Coordinate/radius	Population	Case	RR	LLR	*p* value
1 (38)	458, 553, 588, 521, 214, 497, 251, 573, 239, 116, 95, 22, 198, 171,568, 33, 277, 527, 64, 439, 57, 210, 8, 186, 543, 490, 566, 492, 92, 436, 1, 454, 212, 622, 501, 68, 513, 483, 580, 194	(8.222771 N, 43.571949 E)/197.67 km	926	72	5.21	8.38	< 0.01
2 (2)	337, 376	(6.189488 N, 35.212510 E)/80.81 km	58	10	3.49	5.79	0.52
3 (6)	75, 596, 632, 440, 4, 178	(11.382125 N, 41.702094 E)/76.61 km	131	16	2.48	5.28	0.72
4 (5)	510, 267, 572, 10, 423	(10.160658 N, 38.634847 E)/49.89 km	69	10	5.59	5.02	0.798

### Kriging interpolation of infant mortality

**Fig. 6:**
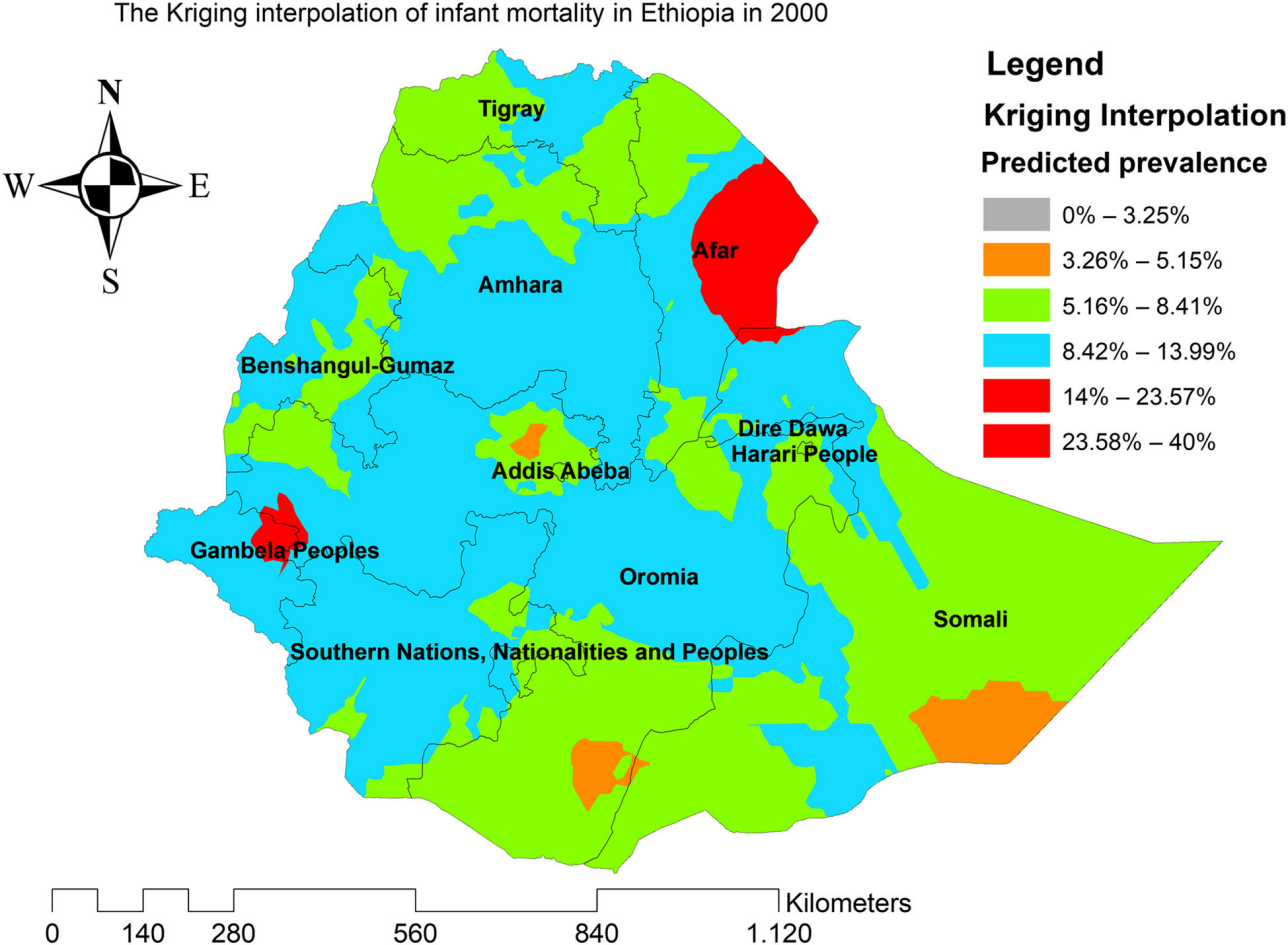
The Kriging interpolation of infant mortality in Ethiopia 2000

**Fig. 7:**
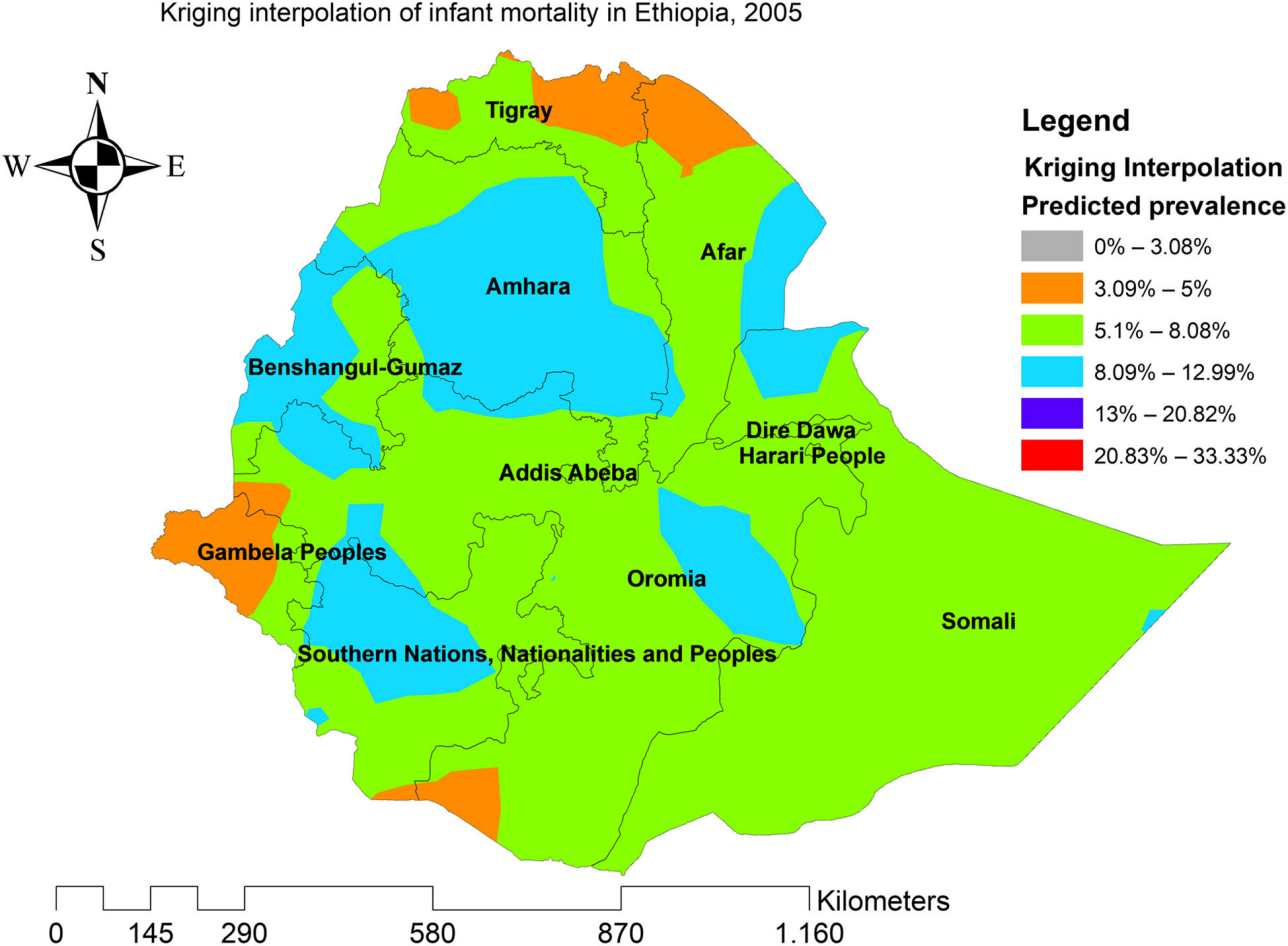
The Kriging interpolation of infant mortality in Ethiopia 2005

**Fig. 8:**
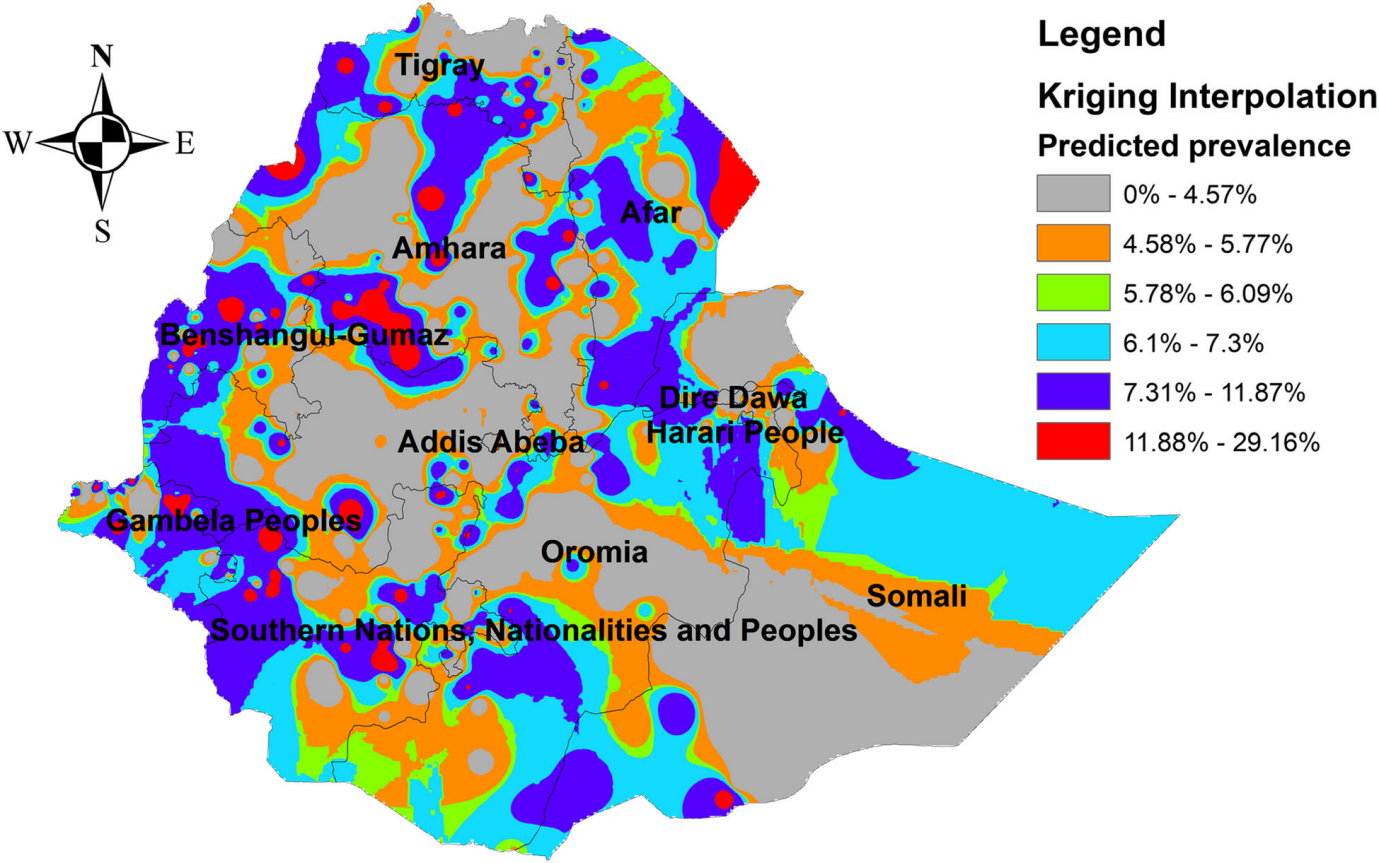
The Kriging interpolation of infant mortality in Ethiopia 2011

**Fig. 9:**
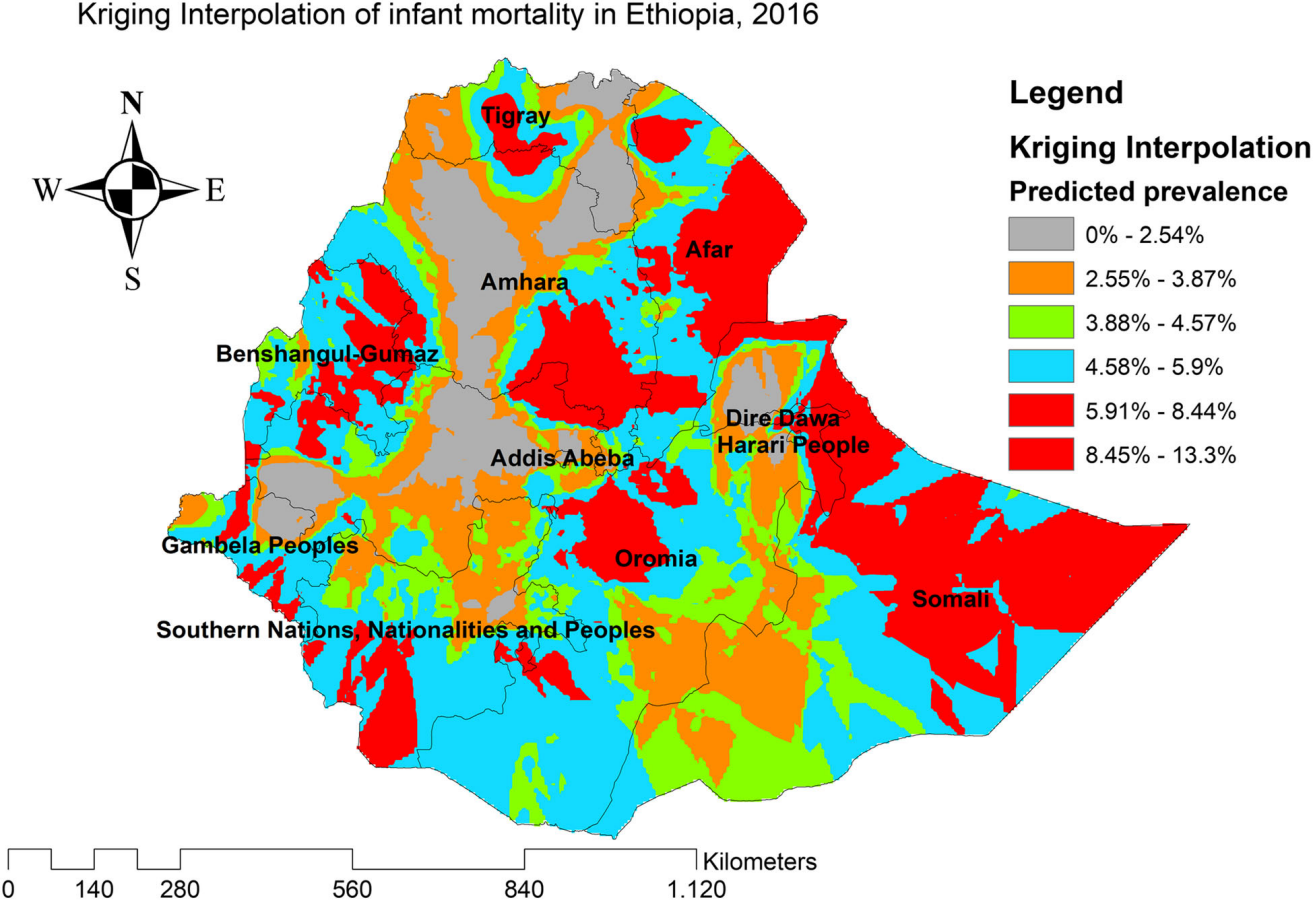
The Kriging interpolation of infant mortality in Ethiopia 2016

In the four EDHS surveys, the ordinary Kriging interpolation technique consistently identified more risky areas of infant mortality in the east Gambella, northeast SNNPRs, border regions of Oromia and Amhara regions, east Afar, and northeast Somali regions of Ethiopia. However, the northwest Tigray, south SNNPRs, south Oromia, southwest Somali, and north Afar regions were identified as the predicted less risky areas of infant mortality (Figs. 6, 7, 8, and 9).

## Discussion

Infant mortality is the most sensitive health indicator that reflects the quality of the health care delivery system and socio-economic progress of the country on the health front [28]. This study found that infant mortality has shown substantial decreases over time in Ethiopia. It is consistent with the study reported in Sub-Saharan Africa (SSA) [21]. This could be attributed to the decrease in the incidence of childhood infectious diseases such as pneumonia, malaria, diarrheal diseases, and sepsis due to integrated management of childhood illness [29], and improved treatment modalities [30]. Another reason might be due to the reduction in poverty, child nutrition practice such as the improved implementation of early breastfeeding and complementary feeding programs, and an increased proportion of health facility delivery that could reduce the incidence of neonatal infections, and complications of labor might contribute for the reduction in infant mortality in Ethiopia [25, 31].

Also, in Ethiopia, the proportion of infants vaccinated for basic childhood vaccines increased over time, and this could decrease the incidence of vaccine-preventable diseases of pneumonia, diphtheria, hepatitis, tetanus, meningitis, polio, diarrhea, and influenza; this might be a possible explanation for the decrease in infant mortality [27, 32]. Also, the establishment of health extension workers (HEWs) to provide preventive and curative services such as basic childhood vaccines, ANC service and treatment for infectious diseases like malaria [26], and improved universal health insurance coverage [33] contributes to the substantial reduction in infant mortality [34].

In Ethiopia, there is substantial residential inequality in infant mortality. This is in line with studies reported in Nigeria [35, 36], Indonesia [37], and Greece [38]. This could be attributed to the disparity in maternal and child health care availability and accessibility and health-seeking behavior across residence [39]. Relatively, urban residents are more aware of maternal and child health services, and the health facilities are easily reachable [40]. Besides, the residential disparity is attributable to the fact that health facilities including private clinics and hospitals are mostly situated in urban areas. The residential inequality significantly increased from 2011 to 2016, this could be due to the increased number of EAs selected in 2016 including the most remote areas such as Somali and Afar regions as these areas were not sampled in 2011 due to security problems. Besides, in the Somali region, in 18 of the 65 selected EAs, listed households were not interviewed for various reasons, such as drought and security problems, and 10 of the 65 selected EAs were not listed due to security reasons [41]. Therefore, the data for the Somali region may not be representative of the region as a whole; this could be the possible justification for this difference.

The spatial patterns of infant mortality were non-random in Ethiopia in all the surveys. In 2000, significant clusters of infant mortality were found in east Afar and at the border areas of Amhara and Oromia regions. This might be due to the limited accessibility of maternal and child health care services such as ANC, health facility delivery, PNC, and childhood vaccinations in the border areas of the country [42]. In 2005, the purely significant clusters of infant mortality were detected in the entire Amhara and north SNNP regions of Ethiopia. The possible explanation might be due to the burden of child malnutrition such as stunting, wasting, and underweight in the Amhara region that was relatively high, as child malnutrition is strongly associated with maternal mortality [43–45]. In 2011, significant clusters of infant mortality were found in the Gambella and SNNP regions of Ethiopia. This might be due to the reason that the border areas are more of pastoralist areas where people did not have permanent residence; due to this, relative health facilities are not accessible and available in these areas. In 2016, significant clusters of infant mortality were identified in the east Oromia and northeast Somali regions. It could be due to these areas are the hotspot areas of common childhood infectious diseases and outbreaks such as diarrheal diseases, measles, pneumonia, and malaria that are assumed to be the leading causes of infant mortality [46].

Despite maternal health service utilization has improved over time, many studies demonstrate the presence of significant inequalities based on urban/rural, education, socio-economic, and regional differences [47]. The spatial disparity could be due to unequal access to health care, the disparity in education and economic factors, and environmental factors across the area [48]. This implies that identifying clusters with high infant mortality is important for prioritizing areas for analysis of cause and planning of remedial actions.

This study had several strengths. First, the study was based on nationally representative large datasets since the DHS statisticians used a standardized tool to collect the data and took samples by stratifying the country into strata to get a representative sample. Second, the estimates of the study were done after the data were weighted for the probability sampling and non-response, to make it representative at national and regional levels: therefore, it can be generalized to birth from reproductive-age women in Ethiopia. Third, the use of GIS and SaTScan statistical tests helped to detect similar and statistically significant hotspot areas of infant mortality practice across the surveys and to design effective public health programs.

The limitations are the SaTScan detect only circular clusters, and irregularly shaped clusters were not detected. Furthermore, the EDHS survey did not incorporate community-level variables like community norm, culture, and beliefs, and medical factors; rather, it relied on mothers or caregivers report and might have the possibility of social desirability and recall bias since infant mortality is not socially acceptable though CSA claims that strong effort was made to minimize it mainly through extensive training of data collectors and recruiting experienced data collectors and supervisors that might underestimate our finding.

## Conclusion

The infant mortality rate has shown a dramatic decrease over the last 16 years in Ethiopia was varied across the residence. This highlights the need to give special attention to rural communities to narrow the urban-rural difference. The spatial distribution of infant mortality was non-random in Ethiopia over the four surveys, and GIS and SaTScan identified significant hotspot areas of infant mortality such as east Oromia and northeast Somali regions. It could help the policymakers and health planners to focus on identified hotspot areas for designing intervention programs working on reducing malnutrition, increases in vaccination, vitamin A, family planning, management of childhood illness, and water and sanitation as these are the main contributors to the improvements in child survival.

## Data Availability

Data is available online, and you can access it from www.measuredhs.com.

## Abbreviations

ANC: Antenatal care
CSA: Central Statistical Agency
DHS: Demographic Health Survey
DM: Diabetes mellitus
EAs: Enumeration areas
EDHS: Ethiopian Demographic and Health Survey
GIS: Geographic Information System
IUGR: Intrauterine growth restriction
LLR: Log-likelihood ratio
LR: Likelihood ratio
MDG: Millennium Development Goals
RR: Relative risk
SNNP: Southern Nations and Nationalities of People
